# Residential Exposure to 50 Hz Magnetic Fields and the Association with Miscarriage Risk: A 2-Year Prospective Cohort Study

**DOI:** 10.1371/journal.pone.0082113

**Published:** 2013-12-03

**Authors:** Qiang Wang, Zhaojin Cao, Yingli Qu, Xiaowu Peng, Shu Guo, Li Chen

**Affiliations:** 1 Department of Environmental Epidemiology, Institute for Environmental Health and Related Product Safety, Chinese Center for Disease Control and Prevention, Beijing, China; 2 Center for Research on Environment and Health, South China Institute of Environmental Sciences, Ministry of Environmental Protection, Guangzhou, Guangdong, China; 3 Chinese Journal of Preventive Medicine, Chinese Medical Association, Beijing, China; Université de Montréal, Canada

## Abstract

**Objective:**

The hypothesis of whether exposure to extremely low-frequency magnetic fields (ELF-MF) may increase miscarriage risk is controversial. A 2-year prospective cohort study was designed to study the association between exposure to 50 Hz magnetic fields (MF) and the miscarriage risk for women residing in the area of the Pearl-River Delta of China.

**Method:**

Two towns with densely distributed power supply constructions were selected as the study sites. From 2010 to 2012, 552 women in the region who were at approximately 8 weeks of gestation or who planned to have a baby within 1 year were selected as candidate subjects. Exposure to MF was estimated by measurements at their front doors and in the alley in front of the subjects’ houses. The average exposure level was used as a cutoff point to define the exposed group. Clinical miscarriage was diagnosed by local obstetricians. Staffs from the local population and family planning service stations were responsible for the follow-up interviews every 2 months.

**Results:**

Four hundred and thirteen pregnant women were selected for the cohort study. The average residential exposure to MF was 0.099 µT. No significantly increased risk of miscarriage was found to be associated with the average front-door exposure (p>0.05). However, miscarriage risk was found to be significantly associated with maximum alley exposure (p=0.001). The relative risk (RR) of miscarriage from maximum alley exposure was 2.35 (95% C.I.: 1.18-4.71). In addition, Cox regression analysis showed that the adjusted hazard ratio of maximum alley exposure for miscarriage was 1.72 (95% C.I.:1.10-2.69).

**Conclusion:**

Although the miscarriage incidence was shown to be positively associated with the maximum alley MF exposure, the association between miscarriage risk and the exposure to MF was not confirmed in the study. The results of this study are of interest concerning MF exposure assessment and pregnancy outcomes.

## Introduction

There have been growing concerns regarding the health impacts of EMF (electromagnetic fields) from high-voltage power lines since the publication of studies on childhood leukemia in 1979 [1–3]. Spontaneous abortion was reported to be associated with the increased use of electrically heated beds and blankets which are sources of ELF-MF (extremely low frequency magnetic fields) exposures [4]. However, this association is quite controversial [5–7]. 

“Miscarriage” or "spontaneous abortion” refers to the loss of a pregnancy without external intervention before 20 weeks of gestation. The causes of miscarriage are quite complicated. Genetic backgrounds and environmental risk factors might interact in the pathology of miscarriage [8]. Given the potentially high public health impact, the World Health Organization (WHO) recommended epidemiological research on the possible link of miscarriage and ELF-MF exposure in 2007 [9].

Because there is no convincing evidence on either side, the question of whether ELF-MF is a possible cause of miscarriage is very controversial. In a population-based cohort study, Li et al. reported that no association between miscarriage risk and the average MF level was found, but miscarriage risk was found to be associated with maximum MF exposure levels [10]. Lee et al. reported in a nested case control study that the miscarriage risk from MF exposure was determined by the metric of exposure. When maximum personal MF intensity was used as the index of exposure, miscarriage was found to be associated with MF exposure. When TWA (time-weighted average) MF intensity was used as the index of exposure, miscarriage was not necessarily associated with MF exposure [11]. In a further prospective substudy, TWA exposure was shown to increase risk when fields were above 0.2µT [12]. In 2007, the WHO conducted a review and concluded that there is limited evidence that miscarriage risk is associated with ELF-MF exposure [13].

Because of the specific “family planning” in China, the public is very worried about the health impacts of EMF exposure on their offspring. “Family planning” is a strategic policy for population control (In brief, it can be explained as “couples are encouraged to have only one child”). In 2009, the Ministry of Environmental Protection (MEP) of China sponsored a project to investigate EMF health effects in the Pearl-River Delta of China. In this paper, we report our findings from a 2-year prospective cohort study on the association between clinical miscarriage risk and residential exposure to 50 Hz MF.

## Materials and Methods

### Site Selection

According to the power supply map offered by the China Southern Power Grid, from May 2009 to April 2010 the electric fields and MF of typical high-voltage power lines and substations (110kV–500kV) were monitored in the Pearl-River Delta of China. Sites for investigating the association between miscarriage and 50Hz ELF exposure level were selected for possessing the following characteristics: 1) The towns had relatively dense distributions of high-voltage power lines or substations, and the monitoring records of MF around the houses next to the power supplies (the distance to the power supply is less than 50 meters) demonstrated levels that were higher than 0.4μT; 2) Among the women residents of child-bearing age, more than 80% were local population; 3) The populations were large enough so that the assessment could be fully supported by a prospective cohort study. 

As mentioned above, two towns in the Pearl-River Delta of China (J.G. and R.H.) were selected as the study sites.

### Study Design

The association between 50Hz MF exposure and miscarriage risk was assessed by a 2-year prospective cohort study. In the estimation of the sampling size, 15% and 2.0 were used as the estimates for the prevalence rate of miscarriage and the relative risk.

### Screening of the Subjects

From May 2010 through May 2012, women at 8 weeks of gestation or more were enrolled as candidate subjects. In addition, women who planned to have a baby within 1 year were also selected as candidate subjects. Pregnant women with a history of thalassemia, sicklemia, glucose-6-phosphate dehydrogenase (G-6-PD) deficiency, thyrotoxicosis, or diabetes were excluded from the cohort. In addition, pregnant women who had lived in their current residences for less than half a year or who had immigrated to other towns more than half a year earlier were excluded from the cohort.

Qualified pregnant women were classified as exposed or controls based on exposure levels of 50 Hz MF at front door or in the alley in front of the subject’s house. The midpoint (P_50_) measurement of MF around each residence was used as the cutoff point for group classification.

The study was approved by the Ethics Committee of Institute for Environmental Health and Related Product Safety, Chinese Center for Disease Control and Prevention. All participants had provided their written informed consent to participate in this study. 

### Selection of the Study Population

The pregnant women were required to register every pregnancy by the local population and family planning service stations (to be briefed as local stations). Local stations were invited as partners and were responsible for selecting pregnant women every 2 weeks. 

### Questionnaires

A 7-part questionnaire was designed to investigate miscarriage risk factors. In brief, the categories were 1) basic information regarding the subjects, including age, education, profession, body weight, height, birthday, date of marriage, date of pregnancy registration, place of birth, residential address, and first year at present residence, among others.; 2) information regarding the women, including menstrual history, personal history of disease, family history of diseases, medication records, income, methods of transportation, and personal areas of residence, among others.; 3) pregnancy investigation, including data on the pregnancy, method of becoming pregnant, clinical symptoms of pregnancy, mood(s), wearing of shield clothing, and medical examination records, among others.; 4) behavior information, including smoking, environmental tobacco smoking (ETS), drinking, feeding of pets, and use of electric appliances, among others.; 5) residence information, including renovations or decorations, furniture, decorating materials, distance from power supplies, radio masts or towers, factories, garbage dumps, or outdoor restaurants; 6) information regarding their professions, including any exposure to chemicals or physical agents; and 7) information regarding the husbands, including age, height, weight, education, profession, personal and family history of chronic diseases, smoking, drinking, and use of electric appliances, among others. 

### Follow-up of Pregnancy Outcomes

Fetal development was checked and diagnosed by local clinical obstetricians. Staff from the local stations were asked to register the status of the fetus and the outcomes of every cohort woman’s pregnancy through the end of the observation period.

### Measurements of 50 Hz MF

Residential exposure to 50 Hz MF was measured by *EFA-300 electric and magnetic field analyzers* (Narda Safety Test Solutions, Pfullingen, Germany). The EFA-300 is validated annually by the National Institute of Metrology P.R.CHINA. In the course of taking the measurements, 50 Hz was selected by the FILTER BANDSTOP of the EFA-300 analyzer. Measurements at the front doors of houses, in the alleys in front of the subjects’ houses, and at public clubs for entertainment were performed during pregnancy on days when the electric power supply loads were relatively high. Measurements at each site were averaged from the monitoring of 5 B fields with each monitoring lasting for at least 16 seconds. All measurements were positioned by global positioning system.

### Statistical Analysis

Epi-info software (CDC, Atlanta, USA) version 3.5.3 was used for data management and data comparison. Data were analyzed with SAS 9.1 for Windows (SAS Institute Inc.,Cary, North Carolina, USA). Cumulative incidence and incidence density were used to describe the distribution of miscarriage prevalence among the subjects. Miscarriage risk was tested by a univariate chi-square analysis. The Cochran-Armitage trend test was performed to check the trends of incidence. Relative risk (RR), attributable risk (AR), and population attributable risk proportion (PAR %) were used to estimate the strength of association between miscarriage and MF exposure. Confounding effects were checked by Cox proportional-hazards regression analysis. A pregnancy without a definite outcome at the end of the observation period was defined as censored data. Age, education, profession, history of abnormal pregnancy, self-reported psychological status during pregnancy, behavior, medication, and exposure to other chemicals or physical agents were used as covariates in the Cox model. No missing data were included in the analysis.

### Quality Control

Unqualified subjects were excluded from the cohort study. The protocol of the study was reviewed and approved by a panel composed of experts from the Ministry of Environmental Protection and the Ministry of Health of China. Relative questionnaires were tested and modified after a pilot survey. Investigators were trained in advance. Data required double entry and were checked by data comparison.

## Results

### Study Population

From May 2010 to May 2012, 552 women of child-bearing age were selected as candidate subjects. Four hundred and forty-nine of the candidates were pregnant during the observation period. Thirty-four pregnant women were excluded from the cohort study because they had lived at their present residences for less than half a year or they had immigrated to reside in other towns more than half a year earlier. In addition, 1 pregnant woman with thalassemia and 1 pregnant woman with G-6-PD disease were not qualified for the study. As mentioned above, 413 pregnant women were selected as the cohort subjects. All subjects were from the Chinese Han population. More than 90% of the pregnant women had lived at their current residences for longer than 5 years. Table 1 summarizes the characteristics of the candidate subjects.

**Table 1:** Pregnancy of Candidate Subjects (N, %).

**Pregnancy Status**	**Town of J.G.**	**Town of R.H.**	**Other Towns**	**Total**
Pregnant	278(50.36%,)	137(24.82%,)	34(6.16%)	449(81.34%)
Not yet pregnant	9(1.63%,)	85(15.40%,)	10(1.81%)	103(18.66%)
<0.5y	1	20	0	/
0.5y-	3	38	3	/
1.0y-	1	26	5	/
≥1.5y	3	1	2	/
Total	286(51.81%,)	222(40.22%,)	44(7.97%)	552(100%)

Note: y, years of observation

### 50 Hz MF Exposure Levels of the Subjects

To assess exposure to 50 Hz MF, 562 measurements were performed in the vicinity of the residences of the subjects. The minimal and maximal monitored B fields were 0.012 µT and 4.260 µT, respectively. Of all 562 measurements, 33.6% (189/562) of measurements were lower than 0.05 µT, 13.3% (75/562) of measurements were higher than 0.4 µT, and 4.6% of measurements were higher than 1.0 µT. The average ELF-MF measurement (median) was 0.099 µT. Table 2 summarizes the measurements of the 50Hz MF.

**Table 2:** 50 Hz Magnetic Fields at Residence of Subjects (B, µT).

**Measurements**	**Min**	**P25**	**Median**	**P75**	**P90**	**Max**
Front door of residence	0.012	0.064	0.098	0.098	0.183	2.04
Alley of residence	0.012	0.099	0.099	0.099	0.305	4.26
Public club	0.012	0.064	0.098	0.098	0.249	2.33

### Grouping of the Subjects

The median level of MF exposure (0.1µT) was used as the cutoff point for group classification. Pregnant women whose exposure levels were higher than 0.1 µT were classified as exposed, and those whose exposure levels were lower than 0.1 µT were classified as the controls. Exposed pregnant women with exposure level higher than 0.4 µT were classified into group B; otherwise, they were classified into group A. Table 3 summarizes the classifications of the pregnant women.

**Table 3:** Classification of the Exposed Women and the Controls (N).

**Group**	**Town of J.G.**	**Town of R.H.**	**Total**
The Controls (<0.1μT)	205	107	312
The Exposed (≥0.1μT)	72	29	101
Group A (0.1μT-<0.4μT)	53	14	67
Group B (0.4μT-4.26μT)	19	15	34

### Characteristics of the Subjects

With chi-square analysis, no significant difference was observed between the exposed women and the controls in age, income, education, place of birth, or history of disease (p>0.05). Table 4 summarizes the characteristics of the subjects by different MF monitoring sites.

**Table 4:** Characteristics of the Pregnant Subjects.

	**Front Door MF Measurements as Exposure Index**			**Maximum Alley MF Measurements as Exposure Index**		
**Characteristics**	The Exposed (%)	Controls (%)	P value	The Exposed (%)	Controls (%)	P value
Age			0.128			0.219
20-	29(43.94)	118(34.01)		48(47.53)	120(38.46)	
25-	33(50.00)	182(52.45)		45(44.55)	155(49.68)	
30-42	4(6.06)	47(13.54)		8(7.92)	37(11.86)	
POB			0.627			0.535
local	62(93.94)	320(92.22)		91(90.10)	274(87.82)	
not local	4(6.06)	27(7.78)		10(9.90)	38(12.18)	
Education			0.056			0.141
Lower than BA	46(69.70)	198(57.06)		66(65.35)	178(57.05)	
BA or higher	20(30.30)	146(42.94)		35(34.65)	134(42.95)	
Income (¥)			0.126			0.900
<30,000	25(37.88)	121(34.87)		36(35.65)	115(36.86)	
30,000-	8(12.12)	79(22.77)		19(18.81)	68(21.79)	
≥50,000	26(39.39)	99(28.53)		32(31.68)	93(29.81)	
unknown	7(10.61)	48(13.83)		14(13.86)	36(11.54)	
Local Residence			0.360			0.254
0.5y-	4(6.06)	24(6.92)		5(4.95)	23(7.37)	
5.0y-	20(30.30)	135(38.90)		33(32.67)	123(39.42)	
≥10.0y	42(63.64)	188(54.18)		63(62.38)	166(53.21)	
History of Abnormal Pregnancy			0.179			0.114
no	44(66.67)	259(74.64)		68(67.33)	235(75.32)	
yes	22(33.33)	88(25.36)		33(32.67)	77(24.68)	
Planned Pregnancy			0.129			0.464
no	5(7.58)	31 (8.93)		7(6.93)	29 (9.29)	
yes	61(92.42)	316(91.07)		94(93.07)	283(90.71)	

Note: BA, bachelor’s degree; POB, place of birth; data were tested by a chi-square analysis.

### Subjects’ Incidence Rates of Miscarriage

When the average front-door measurements of MF were used as the index of exposure, the incidence densities for the exposed women and the controls were 17.78% and 12.53%, respectively. The rate difference was not significant (p>0.05). No dose-response trend was observed in the association between MF exposure and the incidence rate of miscarriage either (p>0.05). When the maximum alley measurements were used as the index of exposure, the incidence densities for the exposed and the controls were 23.29% and 10.11%, respectively. With chi-square analysis, a significant difference was observed in the incidence rate of miscarriage between the exposed women and the controls (χ^2^=7.765, p=0.005). The incidence density of group A (17.66%) was 1.7 times as high as that of the controls (10.11%), and the incidence density of group B (34.19%) was 3.4 times as high as that of the controls. There was a dose-response trend in the association between magnetic field exposure and the incidence of miscarriage (Cochran-Armitage trend test, p<0.01). Table 5 summarizes the incidences of miscarriage among the subjects by MF monitoring site.

**Table 5:** Miscarriage Incidence of the Subjects.

	**Front Door MF Measurements as Exposure Index**					**Maximum Alley MF Measurements as Exposure Index**				
**Group**	**Subjects (N)**	**Observation (PY)**	**Miscarriage (N)**	**CI (%)**	**ID (100PY)**	**Subjects (N)**	**Observation (PY)**	**Miscarriage (N)**	**CI (%)**	**ID (100PY)**
The Controls	347	231.4	29	8.36	12.53	312	207.7	21	6.73	10.11
The Exposed	66	45.0	8	12.12	17.78	101	68.7	16	15.84	23.29*
Group A (0.1μT-<0.4μT)	46	31.0	6	13.04	19.35	67	45.3	8	11.94	17.66
Group B (0.4μT-4.26μT)	20	14.0	2	10.00	14.28	34	13.4	8	23.53	34.19

Note: CI, cumulative incidence; ID, incidence density; PY, person year*, tested by chi-square, p<0.05.

### Strength of the Association between Magnetic Field Exposure and Miscarriage

When the average front-door measurements of MF were used as the index of exposure, the relative risk of miscarriage for the exposed group was 1.4(95% C.I.: 0.6-3.1). When the arithmetic mean of the front-door measurements and the maximum alley measurements were used as the index of exposure, the relative risk of miscarriage for the exposed was 1.9(95% C.I.: 0.9-3.8); however, when the maximum alley measurements of MF were used as the index of exposure, the MF level was moderately associated with miscarriage. The relative risk of miscarriage for the exposed pregnant women was 2.35 (95% C.I.: 1.18-4.71). And the attributable risk of miscarriage was 9.11%. In addition, the population attributable risk proportion was 24.87%.

### Results of Cox Regression Analysis

A chi-square analysis was used to test the association between miscarriage and the other possible risk factors, including health status, behavior, the use of household appliances, indoor chemicals, and outdoor environmental risks. As a result, education, depression, smoking, ETS, history of abnormal pregnancy outcomes, history of diseases specific to women, medical X-ray examination in the past 2 years, and use of folic acid were confounding factors for miscarriage. In addition to the mentioned above risk factors, maternal age, drinking, residential renovations, and townships were adjusted as covariates in the Cox regression analysis.

When the average front-door measurements of MF were used as the index of exposure, MF exposure did not predict miscarriage. After controlling for the confounders, the adjusted hazard ratio of 50 Hz MF exposure was 1.67 (95%C.I.:0.25-11.11); however, when the maximum alley measurements of MF were used as the index of exposure and after controlling for confounders, the Cox model showed that maximum alley exposure to 50 Hz magnetic fields, self-reported depression during pregnancy, and history of abnormal pregnancy increased the risk of miscarriage. The adjusted hazard ratio of magnetic field exposure was 1.72(1.10-2.69). Table 6 summarizes the results of the multivariate Cox regression analysis by MF monitoring site.

**Table 6:** Association between Miscarriage and Exposure to Risk Factors.

	**Front Door MF Measurements as Exposure Index**				**Maximum Alley MF Measurements as Exposure Index**			
**Risk factors**	β	SE	Hazard Ratio (95%C.I.,)	P value	β	SE	Hazard Ratio (95%C.I.,)	P value
Depression (yes vs. no,)	1.381	0.578	3.98(1.28-12.35)	0.017	1.542	0.569	4.68(1.53-14.28)	0.007
History of abnormal pregnancy (yes vs. no,)	2.212	0.468	9.14(3.65-22.87)	<0.001	2.103	0.475	8.19(3.23-20.79)	<0.001
Exposure to 50 Hz MF (per μT increase,)	0.514	0.966	1.67(0.25-11.11)	0.595**^*a*^**	0.543	0.228	1.72(1.10-2.69)	0.017

Note: β, regression coefficient; SE, standard error; exposure to 50 Hz MF in the Cox regression is the measured values at the front door or in the alley in front of the subjects’ houses. Multivariate analysis was conducted using Cox regression analysis; **^*a*^**, the front door MF exposure was retained in the model.

## Discussion

The hypothesis that miscarriage risk is associated with exposure to ELF is still controversial [11–14]. Epidemiological studies on this possible link were recommended in the *2007 WHO Research Agenda for Extremely Low Frequency Fields* [9]. In this study, we attempted to estimate the association between 50 Hz MF exposure and miscarriage risk for women living in the Pearl-River Delta of China through a 2-year cohort study.

Two towns with densely distributed power supply constructions were selected as the sites of the study; however, the intensities of exposure were relatively low. The average exposure level was only approximately 2 times higher than that of the general public (0.05μT). Among all 413 pregnant women, only 34 subjects (8.2%) were exposed to MFs above 0.4µT. Fewer than one-eighth (50 pregnant women) of the subjects lived within100 meters of a power supply. In addition, except for 1 barber and 9 factory laborers, all of the other pregnant women (97.6%) in the cohort had no histories of professional exposure to ELF. 

Due to the China’s current “family planning policy”, approximately 20 percent of the cohort subjects (according to the questionnaires) quit their jobs before pregnancy or at the start of pregnancy for the health of the fetus. Not counting the newly unemployed subjects in the cohort, 29.5% of the pregnant women were not working. These women spent much of their time at home, and chatting in the alley in front of the subjects’ houses and playing mahjong in public clubs are also common activities. Thus, we aimed to assess the association of miscarriage risk with exposure to MF in the alley in front of the subjects’ houses and in clubs. In the course of analysis, we first tried to weight the exposures at the front door, in the alley in front of the subjects’ houses, and at the public club, but found it difficult to assign values to these sites. To interfere with the pregnancies as little as possible, records of daily activities were not required. In addition, due to China’s family planning policy, every pregnancy is treated with great care by the family. To ensure the health of the fetus, no subject agreed to wear the dosimeter during pregnancy. Instead, we separately assessed miscarriage risk by site. 

Due to relatively low levels of MF exposures and the presence of confounders, we cannot conclude definitively that 50 Hz MF is a risk factor for miscarriage; however, we observed apparently contradictory phenomena in connection with this association. The strength of the association of MF exposure with miscarriage risk varied with MF monitoring sites and dose metrics. When the arithmetic mean of the front door and the alley values was included in the Cox model, the MF exposure level was significantly associated with miscarriage risk (α=0.10); however, the lower limit of the hazard ratio was lower than 1.0 (0.9-4.8), and when the measurement at the front door was included in the model, the regression analysis did not show MF exposure to be a risk factor for miscarriage. When the maximum measurement in the alley in front of the subject’s house was used as a variable in the Cox regression model, MF exposure was shown to raise the risk of miscarriage with MF level increase. It’s not known whether this association was related to the women’s custom of sitting on the alley and chatting while selecting vegetables for cooking. 

The results of this study are somewhat similar to those of the population-based prospective cohort study [10] and a sub-study in the nested case-control study led by Lee et al. [11]. Both of these previous studies showed that the link between MF exposure and miscarriage is associated with maximum levels of exposure, but the exposure levels of the subjects in the cohort were slightly lower than those of women residing in the San Francisco area.

Although MF exposure was not time-weighted in this study, such weighting may prove helpful in future studies to determine the strength of the association. Thus, we suggest that it may be more prudent to determine the strength of association.

## Conclusion

The associations of miscarriage between MF exposures were not consistent with all exposure metric by different monitoring sites. We can’t confirm 50 Hz MF as one of possible risks of miscarriage in the cohort study. This study was limited to the Pearl River Delta area of China, and the population was limited to Chinese women. The association between exposure to 50 Hz magnetic fields and miscarriage should be explored in further studies.
